# Mechanical and Thermodynamic Properties of Al_11_(Ce, M)_3_ (M = La, Nd) Phases in Heat-Resistant Aluminum: A First-Principles Calculation Study

**DOI:** 10.3390/ma19040701

**Published:** 2026-02-12

**Authors:** Yihao Wang, Kai Sun, Danlei Zhao

**Affiliations:** 1 School of Mechanics and Aerospace Engineering, Dalian University of Technology, Dalian 116024, China; 2 School of Mechanical Engineering, Dalian University of Technology, Dalian 116024, China

**Keywords:** heat-resistant aluminum alloy, Al-Ce alloy, first-principles calculation, elastic property, thermodynamic property, electronic structure

## Abstract

Aluminum alloys are among the most widely used non-ferrous structural materials in industry, but their insufficient heat resistance severely restricts their application expansion in high-end scenarios, particularly in the aerospace field. As a crucial branch of next-generation heat-resistant aluminum alloys, the Al-Ce series alloys rely on the optimized design of alloying elements to enhance their heat resistance and comprehensive mechanical properties. Based on first-principles calculations using density functional theory (DFT), this study systematically investigated the effects of La and Nd single doping and co-doping on the crystal structure, elastic mechanical properties, lattice dynamics, thermodynamic properties, and electronic structure of the Al_11_Ce_3_ phase. The results demonstrate that all five doped phases exhibit dynamic and thermodynamic stabilities; among them, the Al_11_(Ce, La)_3_ phase shows the highest shear modulus (47.7 GPa), Vickers hardness (8.54 GPa), and Debye temperature (409 K). Furthermore, the synergistic doping of La and Nd can improve the metallicity and ductility of the alloy while maintaining high stiffness. Calculations on electronic properties further reveal the mixed bonding characteristics of Al-RE covalent bonds and metallic bonds, as well as their intrinsic correlation with mechanical property indicators. Our systematic study based on DFT calculations provides theoretical support for regulating the key strengthening phases of Al-Ce-based heat-resistant alloys through rare earth composite microalloying.

## 1. Introduction

Aluminum alloys, characterized by high specific strength and low density, are widely used in aerospace, automotive, and rail transportation sectors. With the rapid advancement of high-tech technologies and products, aluminum alloys are increasingly required to withstand harsh service environments, among which heat resistance is a critical demand [1,2]. For instance, in applications such as the skin and wing panels of hypersonic vehicles, aluminum alloys need to maintain stable performance at 200–400 °C [3,4]. However, traditional aluminum alloys (e.g., 2xxx, 6xxx, and 7xxx series) are typical precipitation-strengthened alloys; when the temperature exceeds 200 °C, their primary strengthening phases (e.g., θ′-Al_2_Cu, β′-Mg_2_Si, and η′-MgZn_2_) tend to coarsen rapidly, leading to a sharp decline in alloy strength [5]. Thus, developing high-heat-resistant aluminum alloys is a pivotal challenge for high-end equipment manufacturing.

Research on heat-resistant aluminum alloys traces back to the 1950s–1960s. Currently, the design of such alloys centers primarily on two alloy systems. The first is the Al-Cu alloy system, with its core design strategy centered on the “coupling of fast-diffusing matrix elements and multiple slow-diffusing additive elements”. By modulating the segregation and diffusion behavior of additive elements, the thermal stability of precipitates is improved [6,7,8]. A typical example is the Al-Cu-Mg-Ag system: via multi-step aging heat treatment, slow-diffusing elements (e.g., Sc, Zr) segregate at interfaces prior to the excessive coarsening of θ′-Al_2_Cu precipitates. This Sc/Zr interfacial segregation not only traps Cu atoms within θ′-Al_2_Cu to preserve its thermal stability but also suppresses the accelerated coarsening of Al_3_Sc precipitates induced by Cu atoms released from θ′-Al_2_Cu dissolution. The second is the Al-TM (transition metal, TM) alloy system, whose design hinges on engineering heat-resistant eutectic microstructures. By incorporating high-melting-point eutectic structures, the mechanical stability of aluminum alloys under elevated temperatures is secured [9,10,11]. However, harmful eutectic phases (e.g., Al_3_Fe) act like large stones mixed into concrete (the Al matrix) during solidification, disrupting the material’s homogeneity and structural continuity. Thus, precise heat treatment is critical to suppressing harmful eutectic phases and facilitating the formation of beneficial precipitates [12,13].

The Al_11_Ce_3_ phase formed by Ce (a rare earth element) and Al has emerged as a core strengthening phase for next-generation heat-resistant aluminum alloys, attributed to its high melting point, low lattice mismatch with the Al matrix, and strong interface bonding. Qi et al. [9] observed via arc melting experiments that Al_11_Ce_3_ forms a fine lamellar structure in Al-based eutectic alloys, with a room-temperature hardness of 4.1–4.3 GPa and low coarsening rate even at 800 °C. Wu and Dunand [14] investigated the microstructure, thermal expansion, and creep properties of the intermetallic compound Al_11_Ce_3_ (the strengthening phase in Al-Ce-based eutectic alloys) and reported that the creep resistance of Al_11_Ce_3_ at 500 °C is comparable to that of L1_2_-Al_3_Sc. Zhang et al. [15] explored the orientation relationship and interface structure between Al_11_Ce_3_ and Al in Al-Ce eutectic alloys; first-principles calculations revealed that the Al_11_Ce_3_/Al interface exhibits chemical adsorption with strong interactions. This strong interface bonding between the strengthening phase and the Al matrix effectively enhances load transfer efficiency, thereby improving material strength. Ding et al. [16] demonstrated via first-principles calculations that the strong hybridization between Al 3*p* orbitals and Ce 4*f* orbitals in Al_11_Ce_3_ is the atomic origin of its high elastic modulus (110 GPa). Recently, Qi et al. found that La and Nd can substitute Ce sites in Al_11_Ce_3_ to form a single Al_11_(Ce, La, Nd)_3_ solid solution, which further reduces the lattice mismatch with the Al matrix and enhances the stability of Al_11_Ce_3_ in the Al matrix. Lv et al. [17] combined first-principles calculations with quasi-harmonic approximation to evaluate the thermodynamic stability of Al_11_RE_3_ (with 17 different rare earth elements) at 0–1000K. Yin et al. [18] calculated the interfacial energy, strain energy, and chemical driving force of the Al_11_Ce_3_/Al interface via first-principles calculations, providing new insights for heat-resistant aluminum alloy design.

Although the chemical composition and crystallographic features of Al_11_Ce_3_ (RE = Ce, La, Nd, etc.) fundamentally govern the macroscopic mechanical properties and thermal stability of the corresponding alloys, systematic comparative investigations into their elastic, thermodynamic, and electronic structure evolution across a compositional gradient, ranging from single- to dual- and triple-RE doping, remain scarce. Most existing research has focused on single-RE variants such as Al_11_Ce_3_, Al_11_La_3_, or Al_11_Nd_3_ [16,19], leaving the composition-structure-property relationships in multi-RE co-doped systems largely unresolved. Important atomic-scale questions thus remain unresolved: for instance, how lattice distortions induced by substituting Ce (atomic radius 101 pm) with larger La (103.2 pm) or smaller Nd (98.3 pm) influence elastic stiffness, and whether hybridization between the *f*/*d* orbitals of multiple RE elements and Al *p* orbitals leads to a synergistic enhancement in bonding strength. Addressing these fundamental issues requires the detailed insights afforded by first-principles calculations.

Herein, we systematically investigate the structural stability, mechanical properties, thermodynamic behaviors, and electronic structures of Al_11_Ce_3_, Al_11_La_3_, and multi-doped phases (Al_11_(Ce, La)_3_, Al_11_(Ce, Nd)_3_, Al_11_(Ce, La, Nd)_3_) using first-principles calculations. This work aims to clarify the strengthening mechanism of multi-RE doping and provide theoretical support for the development of advanced heat-resistant Al-RE alloys.

## 2. Computational Methods and Models

First-principles calculations were performed using the Vienna Ab Initio Simulation Package, version 5.4.4 (VASP 5.4.4) [20] based on density functional theory (DFT). The generalized gradient approximation (GGA) with the Perdew-Burke-Ernzerhof (PBE) functional [21] was adopted to describe the exchange–correlation interaction, and the projector augmented-wave (PAW) method [22] was used to treat ion–electron interactions. Specifically, the recommended PAW potentials are characterized by the following valence electron configurations for each target element: Al (3*s*^2^3*p*^1^), Ce (4*f*^1^5*s*^2^5*p*^6^5*d*^1^6*s*^2^), Nd_3 (5*s*^2^5*p*^6^5*d*^1^6*s*^2^), and La (4*f*^0.0001^5*s*^2^5*p*^6^5*d*^0.9999^6*s*^2^). The Brillouin zone was sampled using a Monkhorst-Pack *k*-point grid [23]. Based on the principle that *k*-point density should be as uniform as possible across all crystal directions, we systematically evaluated the influence of six distinct *k*-point meshes (5 × 1 × 1, 6 × 2 × 2, 7 × 3 × 3, 8 × 3 × 3, 9 × 4 × 3, and 10 × 4 × 3) on the calculated total energy and enthalpies of formation per atom. The results demonstrate that the 8 × 3 × 3 mesh, corresponding to a *k*-point density of 0.03 (2π·Å^−1^), satisfies the convergence requirement, yielding a convergence precision below 0.0005 eV·atom^−1^ for both energy-related metrics. Furthermore, a plane-wave cutoff energy of 350 eV and the optimized *k*-point density were configured to satisfy the convergence criteria of 10^−5^ eV for the self-consistent cycle of electronic steps and <0.01 eV·Å^−1^ for the force convergence of ionic steps. Upon achieving full convergence of both energy and force, the equilibrium lattice parameters of the target structures were extracted.

For orthorhombic crystals, there are nine independent elastic constants (*C_11_*, *C_12_*, *C_13_*, *C_22_*, *C_23_*, *C_33_*, *C_44_*, *C_55_*, and *C_66_*) due to symmetry, which were calculated using the stress–strain method. Phonon dispersion relations were computed via PHONOPY 2.24.0 [24], with the *k*-path generated using the VASPKIT package (version 1.2.5) [25]. Thermodynamic properties were derived based on the quasi-harmonic Debye model using the GIBBS2 package (URL: https://github.com/aoterodelaroza/gibbs2; Access date: 10 June 2024) [26]. Finally, the atomic bonding characteristics in different phases were further analyzed via calculations of partial density of states (PDOS) and electron localization function (ELF) [27].

The theoretical polycrystalline elastic moduli of Al_11_RE_3_ were derived from the nine independent elastic stiffness constants. Two approximation methods were used to calculate polycrystalline moduli: the Voigt method (providing the upper bound) [28] and the Reuss method (providing the lower bound) [29]. For orthorhombic crystals, the shear modulus (*G*) and bulk modulus (*B*) based on Voigt and Reuss approximations are given by:

(1) $$G_{V}=\frac{1}{15}\left(C_{11}+C_{22}+C_{33}\right)-\frac{1}{15}\left(C_{12}+C_{13}+C_{23}\right)+\frac{1}{15}\left(C_{44}+C_{55}+C_{66}\right)$$

(2) $$\frac{1}{G_{R}}=\frac{4}{15}\left(S_{11}+S_{22}+S_{33}\right)-\frac{4}{15}\left(S_{12}+S_{13}+S_{23}\right)+\frac{3}{15}\left(S_{44}+S_{55}+S_{66}\right)$$

(3) $$B_{V}=\frac{1}{9}\left(C_{11}+C_{22}+C_{33}\right)+\frac{2}{9}\left(C_{12}+C_{13}+C_{23}\right)$$

(4) $$\frac{1}{B_{R}}=\left(S_{11}+S_{22}+S_{33}\right)+2\left(S_{12}+S_{13}+S_{23}\right)$$

where the subscripts *V* and *R* denote the Voigt and Reuss approximations, respectively. The arithmetic average of the Voigt and Reuss bounds is referred to as the Voigt-Reuss-Hill (VRH) average [30], which is considered the optimal estimate of the theoretical polycrystalline elastic modulus, and can be expressed as:

(5) $$G=\frac{1}{2}\left(G_{R}+G_{V}\right)$$

(6) $$B=\frac{1}{2}\left(K_{R}+K_{V}\right)$$

The isotropic bulk modulus and shear modulus values for polycrystalline Al_11_RE_3_ are calculated using the above equations. The polycrystalline elastic modulus and Poisson’s ratio can be computed from these values using the relationships:

(7) $$K=\frac{9GB}{3B+G}$$

(8) $$\upsilon =\frac{9B-2G}{2(3B+G)}$$

## 3. Results and Discussion

### 3.1. Lattice Parameters and Solid Solution Characteristics

**Figure 1:**
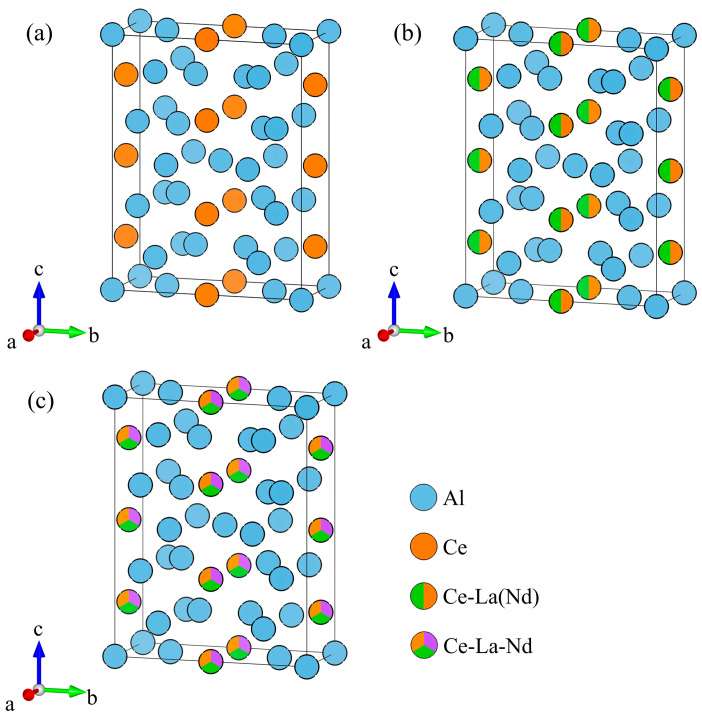
Schematic diagram of Al_11_(Ce, *M*)_3_ (*M* = La, Nd) phases structures. (**a**) Al_11_Ce_3_; (**b**) Al_11_(Ce, La or Nd)_3_; (**c**) Al_11_(Ce, La, Nd)_3_.

Figure 1 presents the atomic structure models of Al_11_Ce_3_, Al_11_La_3_, dual-doped Al_11_(Ce, La)_3_, Al_11_(Ce, Nd)_3_, and triple-doped Al_11_(Ce, La, Nd)_3_. All Al_11_RE_3_ phases belong to the orthorhombic system with space group *Immm*. Each supercell of these phases contains 28 atoms, including 22 Al atoms and 6 RE atoms. In each supercell model, different RE atoms substitute Ce sites in Al_11_Ce_3_, with RE elements occupying the sites at an equiatomic ratio.

For Al_11_(Ce, La)_3_, Al_11_(Ce, Nd)_3_, and Al_11_(Ce, La, Nd)_3_ with binary and ternary RE elements, local chemical effects were not considered, and a completely disordered solid solution model was adopted instead. To obtain the lowest-energy configurations of each doped supercell structure, systematic configuration screening and energy evaluation were conducted. Initially, random structures were constructed via the special quasirandom structure (SQS) method, where the Warren–Cowley short-range order (SRO) [31] parameters were tuned to be as close to zero as possible, corresponding to an ideal random arrangement. These structures were iteratively optimized using the Monte Carlo method, with all calculations implemented in the SQSGENERATOR package (version 0.4.8) [32]. Specifically, the static total energies of 10 SQS models per phase were calculated under the condition of equal molar ratios of RE elements, based on which the most stable atomic configurations of each system were determined (The atomic models and coordination details as shown in Figure S1 and Tables S1–S3).

Table 1 lists the calculated equilibrium lattice parameters of the five Al_11_RE_3_ phases. It is observed that the lattice constants increase regularly with the increase in RE atomic radius: Al_11_La_3_ (La atomic radius: 103.2 pm) exhibits the largest lattice parameters (*a* = 4.423 Å, *b* = 10.183 Å, *c* = 13.135 Å), while Al_11_Ce_3_ (Ce atomic radius: 101 pm) shows the smallest (*a* = 4.359 Å, *b* = 10.011 Å = 12.799 Å). The lattice parameters of multi-doped phases (Al_11_(Ce, La)_3_, Al_11_(Ce, Nd)_3_, Al_11_(Ce, La, Nd)_3_) lie between the two single-RE phases, and the unit cell volume follows the “mixing rule”, reflecting tunability under the chemical size effect. This result is consistent with the experimental observation by Qi et al. [9], i.e., multi-RE forms a continuous solid solution on the RE sublattice of Al_11_RE_3_ without obvious phase separation, which also confirms the limitation of Thermo-Calc thermodynamic predictions.

To evaluate the formation feasibility of Al_11_RE_3_ phases, the enthalpy of formation (Δ*H*) was calculated as a key parameter for assessing the thermodynamic stability of the composite structures. Δ*H* represents the energy released when the constituent elemental monomers combine to form the compound, and its calculation formula is as follows:

(9) $$∆H=\frac{E\left({Al}_{11}{\left(nCe,mLa,lNd\right)}_{3}\right)-\left[22E\left(Al\right)+3nE\left(Ce\right)+3mE\left(La\right)+3lE\left(Nd\right)\right]}{28}$$

where *E*(Al_11_(*n*Ce, *m*La, *l*Nd)_3_) denotes the total energy of the supercell containing 22, *n*, *m*, and *l* atoms of Al, Ce, La, and Nd, respectively. *E*(Al), *E*(Ce), *E*(La), and *E*(Nd) represent the per-atom energies of the elements Al, Ce, La, and Nd in their respective equilibrium bulk phase.

**Figure 2:**
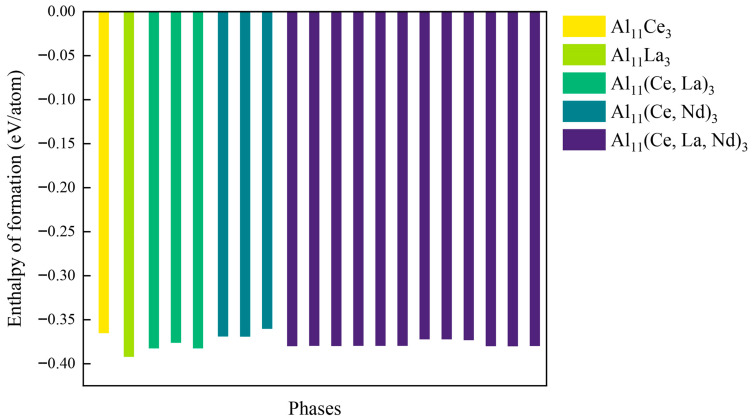
The calculated enthalpies of formation for different Al_11_(Ce, M)_3_ (M = La, Nd) phases.

Figure 2 shows the calculated values of Δ*H* corresponding to all Al_11_RE_3_ phase. To further explore the inherent regularity of these doped systems, partial SQS configurations of Al_11_(Ce, La)_3_, Al_11_(Ce, Nd)_3_, and Al_11_(Ce, La, Nd)_3_ were utilized to access their common energetic features. All values of Δ*H* are negative, indicating the spontaneous formation capability of these phases. A larger absolute value of formation enthalpy corresponds to higher chemical stability. Compared with Al_11_Ce_3_, Al_11_La_3_ and multi-doped phases (Al_11_(Ce, La)_3_, Al_11_(Ce, Nd)_3_, Al_11_(Ce, La, Nd)_3_) exhibit lower formation enthalpies, demonstrating that single or co-doping with La and Nd can significantly enhance the stability of Al_11_RE_3_ and improve its resistance to decomposition.

### 3.2. Elastic Properties

Table 2 summarizes the independent elastic constants (*C*_ij_), bulk modulus (*B*), shear modulus (*G*), Young’s modulus (*K*), Pugh ratio (*B*/*G*) [33], Poisson’s ratio (*ν*, a parameter related to the bonding characteristics of solids), Vickers hardness (*H*_V_) [34], and Debye temperature (*Θ*_D_) for each phase, where *B*, *G*, and *K* were derived using the Voigt-Reuss-Hill averaging scheme. In terms of overall stiffness, the Young’s modulus *K* decreases in the order: Al_11_(Ce, La)_3_ > Al_11_(Ce, La, Nd)_3_ > Al_11_(Ce, Nd)_3_ > Al_11_Ce_3_ > Al_11_La_3_. Both the shear modulus *G* and *H*_V_ follow the same trend as *K*, indicating that the chemical environment formed by Ce-La co-doping can significantly enhance the shear strength and hardness of the phase.

According to the Pugh criterion, the *B*/*G* ratio serves as a key indicator for evaluating the intrinsic brittleness and ductility of materials: a value greater than 1.75 suggests ductility behavior, whereas a value below 1.75 indicates brittleness [35]. As listed in Table 2, Al_11_La_3_ exhibits ductility with *B*/*G* ≈ 1.768, while Al_11_Ce_3_ lies near the brittle–ductile transition with *B*/*G* ≈ 1.749. In contrast, the three multi-RE doped phases exhibit lower *B/G* values, indicating a tendency toward brittle behavior.

**Figure 3:**
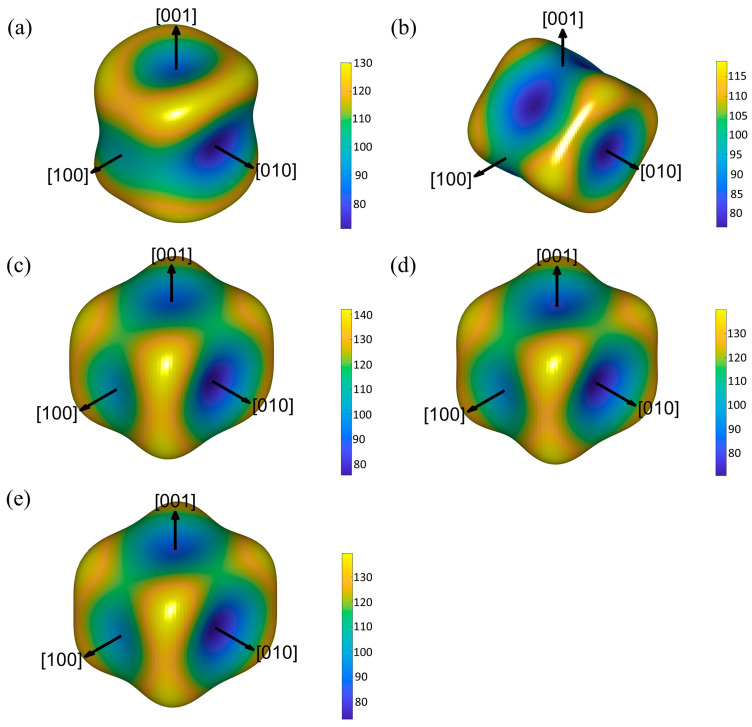
The 3D Young’s modulus *K* curved surfaces of (**a**) Al_11_Ce_3_, (**b**) Al_11_La_3_, (**c**) Al_11_(Ce, La)_3_, (**d**) Al_11_(Ce, Nd)_3_, and (**e**) Al_11_(Ce, La, Nd)_3_, respectively.

Figure 3 displays the 3D Young’s modulus surfaces of these Al-RE phases. Al_11_Ce_3_ exhibits the most significant elastic anisotropy; however, after substituting Ce with La/Nd to form Al_11_La_3_, dual-doped Al_11_(Ce, Nd)_3_/Al_11_(Ce, La)_3_, and triple-doped Al_11_(Ce, La, Nd)_3_, the elastic anisotropy decreases significantly. This characteristic is consistent with the calculation results of the universal elastic anisotropy index *A*^U^ [35], which follows the order Al_11_Ce_3_ > Al_11_(Ce, Nd)_3_ > Al_11_(Ce, La, Nd)_3_ > Al_11_(Ce, La)_3_ > Al_11_La_3_. This indicates that co-doping with La and Nd can alleviate the elastic anisotropy of Al_11_RE_3_ to a certain extent.

### 3.3. Lattice Dynamics and Thermodynamic Properties

**Figure 4:**
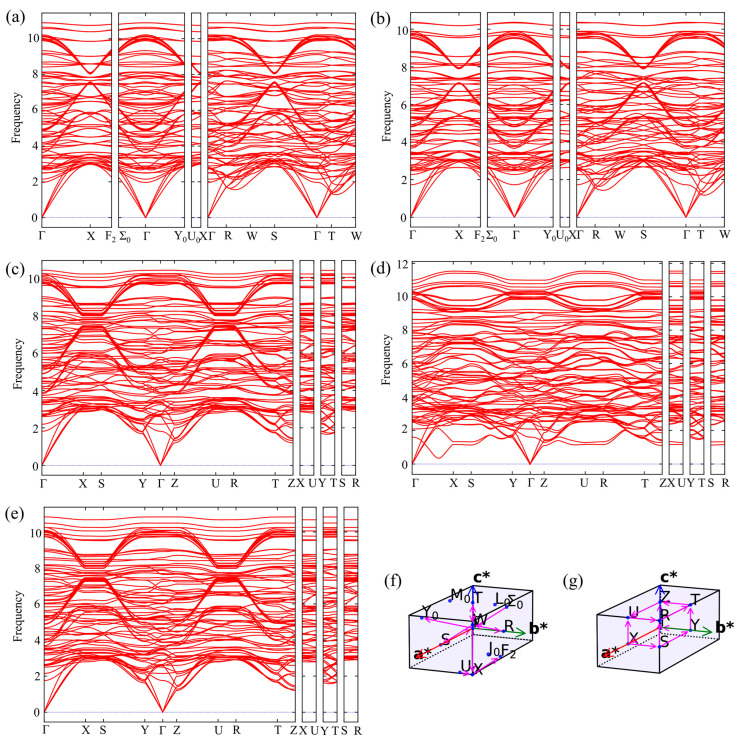
Phonon dispersion curves calculated along the connection direction of high symmetry points in the Brillouin zone of (**a**) Al_11_Ce_3_, (**b**) Al_11_La_3_, (**c**) Al_11_(Ce, La)_3_, (**d**) Al_11_(Ce, Nd)_3_, and (**e**) Al_11_(Ce, La, Nd)_3_. (**f**) Schematic Brillouin zone diagrams corresponding to Al_11_Ce_3_ and Al_11_La_3_. (**g**) Schematic Brillouin zone diagrams corresponding to Al_11_(Ce, La)_3_, Al_11_(Ce, Nd)_3_, Al_11_(Ce, La, Nd)_3_.

Figure 4 presents the phonon dispersion curves of the five phases along the high-symmetry paths in the Brillouin zone. For all supercell structures, no imaginary frequencies are observed in the phonon spectra of the other four phases, confirming their dynamic stability. The Debye temperature (*Θ*_D_) of each phase was calculated from the phonon spectrum, and *Θ*_D_ shows a good positive correlation with modulus and hardness. The *Θ*_D_ values follow the order: Al_11_(Ce, La)_3_ > Al_11_(Ce, La, Nd)_3_ > Al_11_(Ce, Nd)_3_ Al_11_Ce_3_ > Al_11_La, which explains the advantage of Ce-La co-doped phases in high-temperature acoustic stiffness and hardness.

To further compare their stability at finite temperatures, the Gibbs free energy of formation for Al_11_RE_3_ was calculated as follows:

(10) $$∆G_{f}=\frac{G_{f}\left({Al}_{11}{\left(nCe,mLa,lNd\right)}_{3}\right)-\left[22G_{f}\left(Al\right)+3nG_{f}\left(Ce\right)+3mG_{f}\left(La\right)+3lG_{f}\left(Nd\right)\right]}{28}$$

*G_f_*(Al_11_(*n*Ce, *m*La, *l*Nd)_3_), *G_f_*(Al), *G_f_*(La), and *G_f_*(Nd) are the per-atom Gibbs free energies of the Al_11_RE_3_ compound and constituent elements (Al, La, Nd) in their stable states, respectively. The Gibbs free energy at a specific temperature *T* was obtained by minimizing Helmholtz free energy *F*(*T*, *V*) [24]:

(11) $$G_{f}\left(T,\;p\right)=\underset{V}{\min}\left[F\left(T,\;V\right)+pV\right]$$

(12) $$F\left(T,\;V\right)=E\left(V\right)+F_{vib}\left(V,\;T\right)$$

Here, *E*(*V*) is the static energy at 0 K without zero-point vibrational energy. *F*_vib_(*V*, *T*) represents the vibrational free energy contribution based on the quasi-harmonic Debye model.

**Figure 5:**
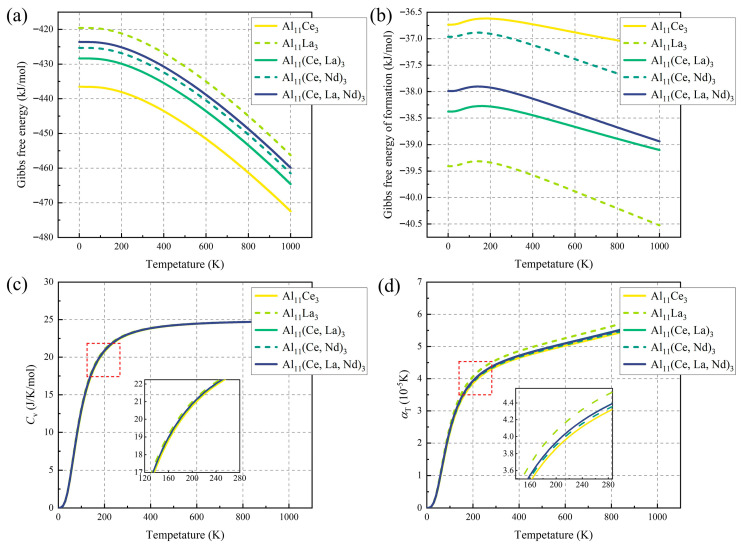
Relation curves of (**a**) Gibbs free energy, (**b**) entropy, (**c**) constant-pressure heat capacity, and (**d**) coefficient of thermal expansion as a function of temperature for Al_11_(Ce, M)_3_ (M = La, Nd) phases.

Figure 5a shows that the Gibbs free energy (*G_f_*) of all phases decreases monotonically with increasing temperature (0–1000 K), attributed to the contribution of thermal vibration entropy; however, the decreasing rate of *G_f_* for multi-doped phases is slower. Figure 5b illustrates the variation in Δ*G_f_* for the five phases: Δ*G_f_* decreases with increasing temperature for all phases, indicating that higher temperatures favor phase formation. Meanwhile, compared with Al_11_Ce_3_, the other four phases exhibit lower Δ*G_f_*, with the formation tendency following the order Al_11_La_3_ > Al_11_(Ce, La)_3_ > Al_11_(Ce, La, Nd)_3_ > Al_11_(Ce, Nd)_3_ > Al_11_Ce_3_.

Figure 5c reveals that the constant-pressure heat capacity (*C_V_*) of all phases first increases and then plateaus with increasing temperature, approaching the Dulong-Petit limit (~25 J/(mol·K)) at 800 K. Both dual-doped phases (Al_11_(Ce, Nd)_3_, Al_11_(Ce, La)_3_) and the triple-doped phase (Al_11_(Ce, La, Nd)_3_) exhibit lower CV and high modulus, indicating good high-temperature dimensional stability. Figure 5d shows the thermal expansion coefficient (α) of each phase: the α of Al_11_(Ce, Nd)_3_ is only higher than that of Al_11_Ce_3_, followed by Al_11_(Ce, La, Nd)_3_ and Al_11_(Ce, La)_3_. This suggests that compared with Al_11_La_3_, dual/multi-RE co-doping can reduce lattice distortion between the precipitate and Al matrix, achieving low interface mismatch between the precipitate and matrix.

### 3.4. Electronic Structure Analysis

**Figure 6:**
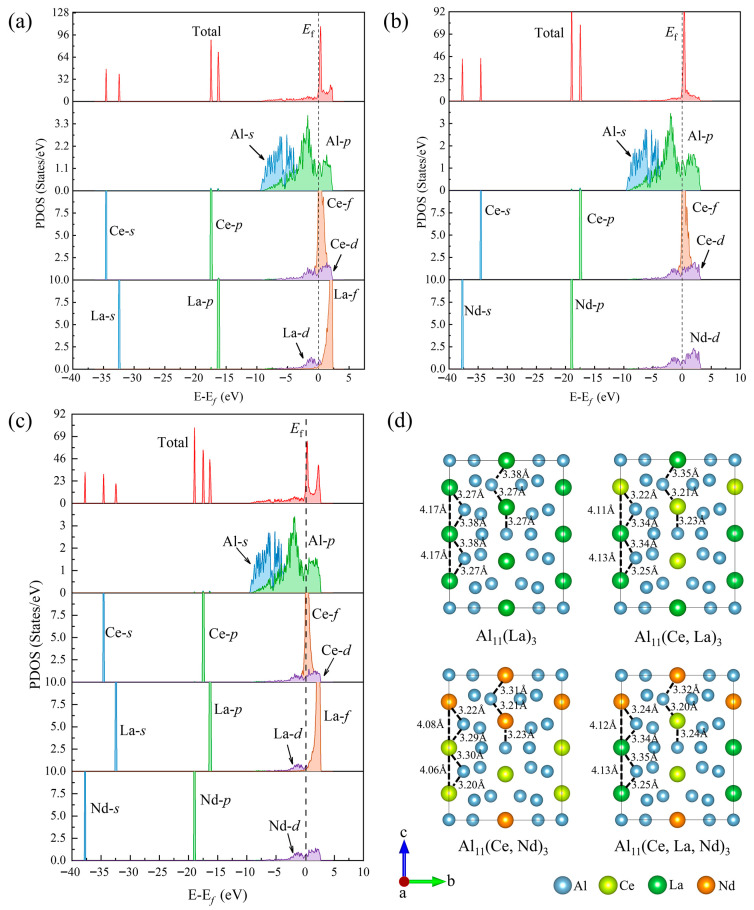
Partial density of states (PDOS) for (**a**) Al_11_(Ce, La)_3_, (**b**) Al_11_(Ce, Nd)_3_, and (**c**) Al_11_(Ce, La, Nd)_3_, respectively. (**d**) Atomic bonding after relaxing cell structure.

The macro-properties of materials (e.g., strength, hardness, ductility/brittleness, stability) are closely related to their electronic structures [36,37,38,39,40]. To clarify the micro-mechanism of La/Nd doping on the structural and elastic properties of Al_11_Ce_3_, the electronic structures of doped systems were analyzed to investigate the solid solution behavior of La and Nd in Al_11_Ce_3_ at the electronic level (Figure 6). Figure 6a–c shows the partial density of states (PDOS) of Al_11_(Ce, La)_3_, Al_11_(Ce, Nd)_3_, and Al_11_(Ce, La, Nd)_3_, respectively, with the dashed line representing the Fermi level (energy = 0). All five phases exhibit non-zero DOS near the Fermi level, confirming that they are metallic materials.

For La-Ce doped phases, electrons are mainly distributed in the energy range of −35~5 eV. In the −35~−30 eV range, the bonding electrons are primarily contributed by the *s* orbitals of La and Ce. In the −20~−15 eV range, the bonding electrons are dominated by the *p* orbitals of La and Ce. Since these two ranges are far from the Fermi level, they have little effect on the system properties. For the Nd-Ce doped and Nd-La-Ce multi-doped systems, the *s* and *p* orbitals of Nd are located at lower energy levels than those of Ce, and no hybridization peaks are observed between Ce-La, Ce-Nd, or Ce-La-Nd, indicating weak chemical bonding between these elements. This result is also verified by the ELF calculations of each phase (Figure S2).

For the three multi-doped phases, the bonding electrons near the Fermi level are mainly contributed by the *p* orbitals of Al, and significant hybridization exists between the *f* orbitals of Ce and the *p* orbitals of Al in this region, indicating strong chemical bonding between Al and Ce. In contrast, the hybridization between the *d* orbitals of La/Nd and *p* orbitals of Al is weak, implying weak chemical bonding between La/Nd and Al atoms. Additionally, by observing the atomic bond lengths after lattice relaxation in Figure 6d, it is found that in Al_3_La, the La-La bond length (4.17 Å) is longer than the RE-RE bond lengths in other dual/triple-doped Al_3_RE phases, indicating weaker bonding. Meanwhile, the La-Al bond length in Al_3_La is also longer than the RE-Al bond lengths at the same positions in the other three phases, further confirming the relatively weak chemical bonding between La and the Al matrix. This explains why the elastic modulus and hardness of Al_11_La_3_ are lower than those of the other three multi-doped phases. In contrast, the RE-Al and RE-RE bond lengths in Al_11_(Ce, La)_3_, Al_11_(Ce, Nd)_3_, and Al_11_(Ce, La, Nd)_3_ are close to each other, indicating similar mechanical properties of these phases.

## 4. Conclusions

Based on first-principles DFT calculations, we have systematically examined how single and co-doping with La and Nd alter the crystal structure, elastic properties, lattice dynamics, thermodynamics, and electronic structure of the Al_11_Ce_3_ phase. The principal findings are as follows:

(1) All five Al_11_RE_3_ (RE = Ce, La, Nd) phases satisfy the criteria for elastic and dynamic stability. The unit cell volume increases with the atomic size of RE, and the volumes of multi-RE doped phases lie between the two single-RE phases.
(2) In terms of mechanical properties, Al_11_(Ce, La)_3_ exhibits the highest shear modulus, Young’s modulus, and Vickers hardness, corresponding to the highest Debye temperature. Meanwhile, multi-RE (La, Nd) doping can maintain high stiffness while enhancing metallicity and ductility.
(3) Thermodynamic calculations at finite temperatures reveal that the introduction of La and its synergistic co-doping with Ce and Nd substantially enhances the phase formation tendency. Moreover, the dual- and triple-RE doped phases combine lower heat capacity coupled with higher moduli, indicating superior high-temperature dimensional stability.
(4) Electronic structure calculations show that Ce-La co-doping strengthens directional bonding and shear strength, while La-Nd incorporation enhances the metallic character near the Fermi level and improves ductility. These insights provide theoretical guidance for the design of rare-earth composite microalloying and precipitation-strengthening engineering.

## Figures and Tables

**Table 1:** The calculated equilibrium lattice parameters *a*, *b*, and *c* (in unite of Å); *α, β*, and *γ* (in unite of °); and volume of *V* (in unite of Å^3^) for different Al_11_(Ce, M)_3_ (M = La, Nd) phases.

Phases	*a*	*b*	*c*	*α*	*β*	*γ*	*V*
Al_11_Ce_3_	4.359	10.011	12.799	90	90	90	558.567
Al_11_La_3_	4.423	10.183	13.135	90	90	90	591.571
Al_11_(Ce, La)_3_	4.372	10.107	13.018	90	90	90	575.179
Al_11_(Ce, Nd)_3_	4.357	10.082	12.885	90	90	90	565.943
Al_11_(Ce, La, Nd)_3_	4.369	10.106	12.990	90	90	90	573.572

**Table 2:** Elastic constants *C_ij_*, bulk modulus *B*, shear modulus *G*, Young’s modulus *K*, *B*/*G* value, Poisson’s ratio *υ*, Cauchy pressure, Vickers hardness *H*_V_, Debye temperature *Θ*_D_, and Universal elastic anisotropy *A*^U^ of Al_11_(Ce, M)_3_ (M = La, Nd) phases.

Phases	Al_11_Ce_3_	Al_11_La_3_	Al_11_(Ce, La)_3_	Al_11_(Ce, Nd)_3_	Al_11_(Ce, La, Nd)_3_
*C*_11_/GPa	133.882	126.4	132.312	128.689	129.506
*C*_12_/GPa	59.028	50.069	54.024	57.758	55.493
*C*_13_/GPa	47.09	40.368	44.021	46.444	45.309
*C*_22_/GPa	110.478	107.238	109.233	108.944	108.435
*C*_23_/GPa	58.012	49.775	52.459	56.17	54.019
*C*_33_/GPa	118.466	116.097	118.412	115.264	115.707
*C*_44_/GPa	61.721	53.268	52.887	54.616	52.948
*C*_55_/GPa	66.096	29.049	60.877	60.062	59.533
*C*_66_/GPa	37.744	52.773	65.616	61.278	62.962
*B*/GPa	76.684	69.968	73.333	74.763	73.603
*G*/GPa	43.862	39.574	47.716	45.597	46.131
*K*/GPa	110.515	99.889	117.634	113.681	114.476
*υ*	0.26	0.262	0.233	0.247	0.241
*B/G*	1.748	1.768	1.537	1.64	1.596
*H*_v_/GPa	6.451	5.785	8.536	7.418	7.828
Universal elastic anisotropy	0.61	0.37	0.47	0.56	0.51
*Θ*_D_/K	391.3	376.3	409.4	397.5	401.4

## Data Availability

The original contributions presented in this study are included in the article/Supplementary Material. Further inquiries can be directed to the corresponding authors.

## Supplementary Materials

The following supporting information can be downloaded at https://www.mdpi.com/article/10.3390/ma19040701/s1, Figure S1: Atomic models for Al_11_(Ce, La)_3_, Al_11_(Ce, Nd)_3_, and Al_11_(Ce, La, Nd)_3_ phases with lowest static energy; Figure S2: The electron localization function (ELF) of (a) Al_11_Ce_3_, (b) Al_11_La_3_, (c) Al_11_(Ce, La)_3_, (d) Al_11_(Ce, Nd)_3_, and (e) Al_11_(Ce, La, Nd)_3_, respectively; Table S1: Fractional atomic coordinates listed herein are referenced to the lowest-static-energy supercell of Al_11_(Ce, La)_3_ special quasirandom structures (SQS), for which the lattice constants are *a* = 4.421 Å, *b* = 10.101 Å, and *c* = 13.128 Å; Table S2: Fractional atomic coordinates listed herein are referenced to the lowest-static-energy supercell of Al_11_(Ce, Nd)_3_ special quasirandom structures (SQS), for which the lattice constants are *a* = 4.421 Å, *b* = 10.101 Å, and *c* = 13.128 Å; Table S3. Fractional atomic coordinates listed herein are referenced to the lowest-static-energy supercell of Al_11_(Ce, La, Nd)_3_ special quasirandom structures (SQS), for which the lattice constants are *a* = 4.421 Å, *b* = 10.101 Å, and *c* = 13.128 Å.
