# Supplementary material for: Mechanical and Thermodynamic Properties of Al11(Ce, M)3 (M = La, Nd) Phases in Heat-Resistant Aluminum: A First-Principles Calculation Study
Source: Materials (Basel). 2026 Feb 12;19(4):701. doi: 10.3390/ma19040701 (PMC12941715; doi:10.3390/ma19040701)
Supplement: Supplementary file 1 [file materials-19-00701-s001.zip › materials-4076143-supplementary.pdf]

materials

Supplementary material

Mechanical and Thermodynamic Properties of $Al_{11}(Ce, M)_3$ (M = La, Nd) Phases in Heat-Resistant Aluminum: A First-Principles Calculation Study

Yihao Wang [1], Kai Sun [1,*] and Danlei Zhao [2,*]

[1] School of Mechanics and Aerospace Engineering, Dalian University of Technology, Dalian 116024, China
[2] School of Mechanical Engineering, Dalian University of Technology, Dalian 116024, China
* Correspondence: sunkai@dlut.edu.cn (K.S.); zhaodanlei@dlut.edu.cn (D.Z.)

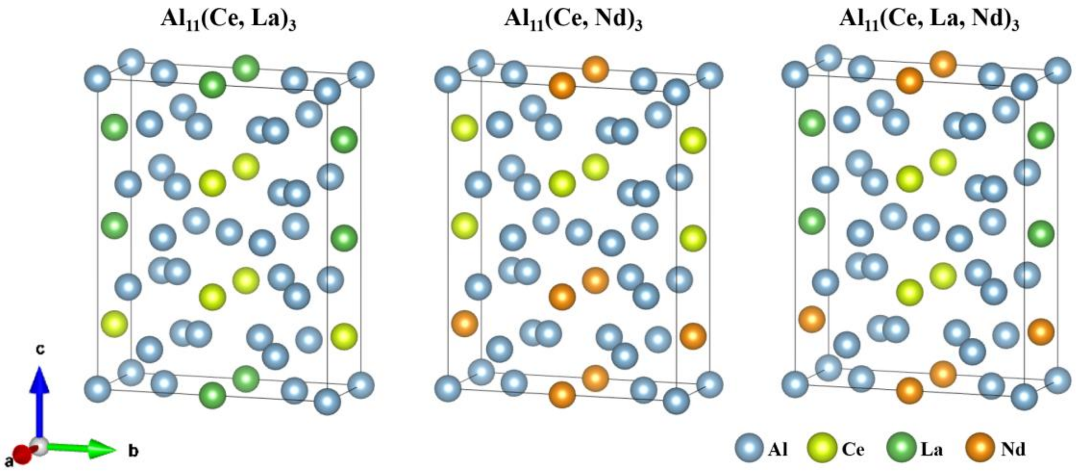

Figure S1. Atomic models for $Al_{11}(Ce, La)_3$, $Al_{11}(Ce, Nd)_3$, $Al_{11}(Ce, La, Nd)_3$ phases with lowest static energy.

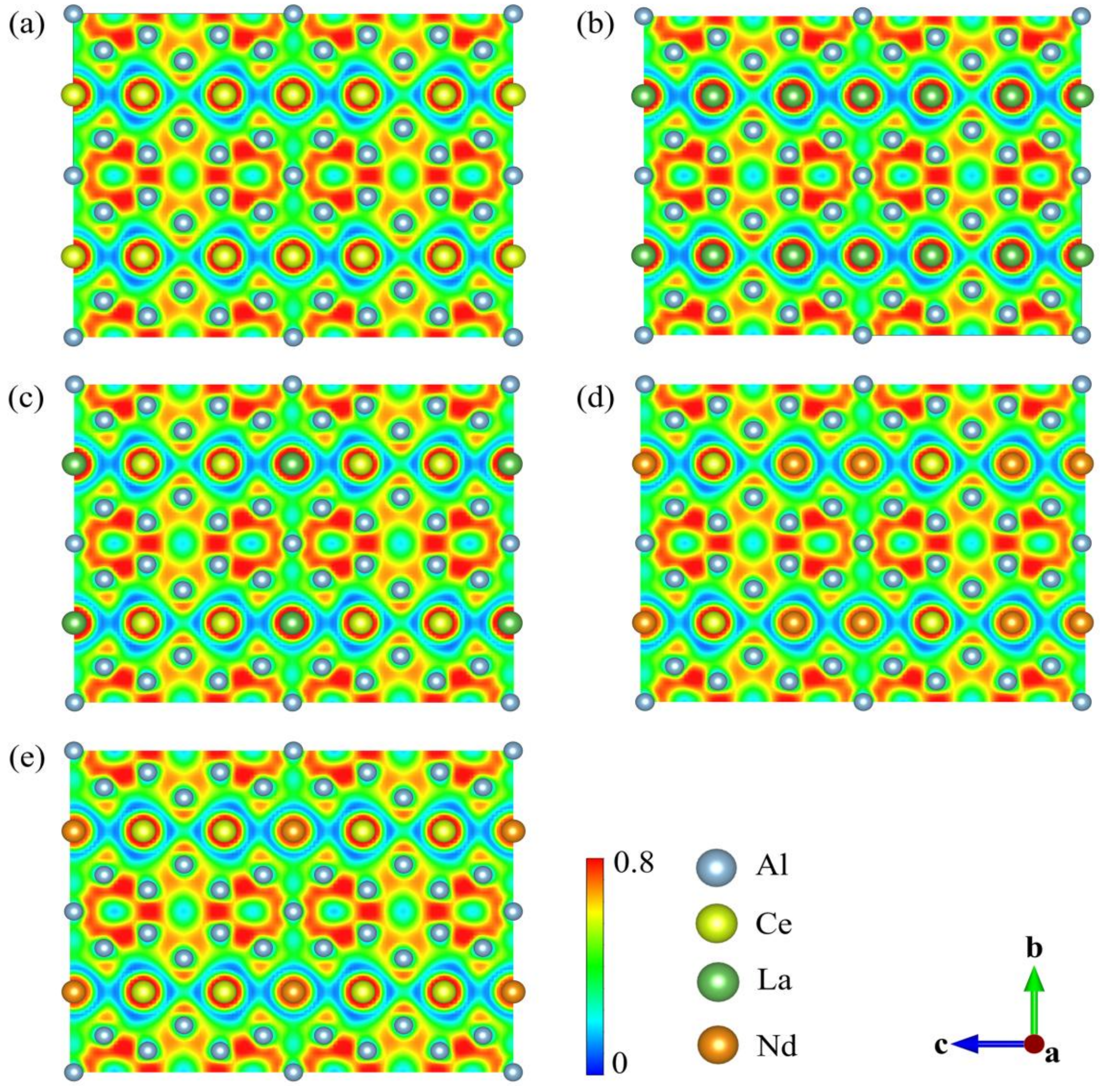

Figure S2. The electron localization function (ELF) of (a) $Al_{11}Ce_3$, (b) $Al_{11}La_3$, (c) $Al_{11}(Ce, La)_3$, (d) $Al_{11}(Ce, Nd)_3$ and (e) $Al_{11}(Ce, La, Nd)_3$, respectively.

Table S1. Fractional atomic coordinates listed herein are referenced to the lowest-static-energy supercell of $Al_{11}(Ce, La)_3$ special quasirandom structures (SQS), for which the lattice constants are a = 4.421 Å, b = 10.101 Å, and c = 13.128 Å.

Atomic coordinates			Elements
0.000	0.500	0.317	Ce1
0.000	0.500	0.683	Ce2
0.500	0.000	0.183	Ce3
0.500	0.000	0.817	La1
0.000	0.500	0.000	La2
0.500	0.000	0.500	La3
0.000	0.133	0.334	Al1
0.000	0.867	0.666	Al2
0.000	0.867	0.334	Al3
0.000	0.133	0.666	Al4
0.500	0.633	0.834	Al5
0.500	0.367	0.166	Al6
0.500	0.367	0.834	Al7
0.500	0.633	0.166	Al8
0.000	0.226	0.137	Al9
0.000	0.774	0.863	Al10

https://doi.org/10.3390/ma19040701

0.000	0.774	0.137	Al11
0.000	0.226	0.863	Al12
0.500	0.726	0.637	Al13
0.500	0.274	0.363	Al14
0.500	0.274	0.637	Al15
0.500	0.726	0.363	Al16
0.000	0.284	0.500	Al17
0.000	0.716	0.500	Al18
0.500	0.784	0.000	Al19
0.500	0.216	0.000	Al20
0.000	0.000	0.000	Al21
0.500	0.500	0.500	Al22

Table S2. Fractional atomic coordinates listed herein are referenced to the lowest-static-energy supercell of $Al_{11}(Ce, Nd)_3$ special quasirandom structures (SQS), for which the lattice constants are a = 4.421 Å, b = 10.101 Å, and c = 13.128 Å.

Atomic coordinates			Elements
0.000	0.500	0.317	Nd1
0.500	0.000	0.183	Nd2
0.000	0.500	0.000	Nd3
0.000	0.500	0.683	Ce1
0.500	0.000	0.817	Ce2
0.500	0.000	0.500	Ce3
0.000	0.133	0.334	Al1
0.000	0.867	0.666	Al2
0.000	0.867	0.334	Al3
0.000	0.133	0.666	Al4
0.500	0.633	0.834	Al5
0.500	0.367	0.166	Al6
0.500	0.367	0.834	Al7
0.500	0.633	0.166	Al8
0.000	0.226	0.137	Al9
0.000	0.774	0.863	Al10
0.000	0.774	0.137	Al11
0.000	0.226	0.863	Al12
0.500	0.726	0.637	Al13
0.500	0.274	0.363	Al14
0.500	0.274	0.637	Al15
0.500	0.726	0.363	Al16
0.000	0.284	0.500	Al17
0.000	0.716	0.500	Al18
0.500	0.784	0.000	Al19
0.500	0.216	0.000	Al20
0.000	0.000	0.000	Al21
0.500	0.500	0.500	Al22

Table S3. Fractional atomic coordinates listed herein are referenced to the lowest-static-energy supercell of $Al_{11}(Ce, La, Nd)_3$ special quasirandom structures (SQS), for which the lattice constants are a = 4.421 Å, b = 10.101 Å, and c = 13.128 Å.

Atomic coordinates			Elements
0.500	0.000	0.183	Nd1
0.000	0.500	0.000	Nd2
0.000	0.500	0.317	Ce1
0.000	0.500	0.683	Ce2
0.500	0.000	0.817	La1
0.500	0.000	0.500	La2
0.000	0.133	0.334	Al1

https://doi.org/10.3390/ma19040701

0.000	0.867	0.666	Al2
0.000	0.867	0.334	Al3
0.000	0.133	0.666	Al4
0.500	0.633	0.834	Al5
0.500	0.367	0.166	Al6
0.500	0.367	0.834	Al7
0.500	0.633	0.166	Al8
0.000	0.226	0.137	Al9
0.000	0.774	0.863	Al10
0.000	0.774	0.137	Al11
0.000	0.226	0.863	Al12
0.500	0.726	0.637	Al13
0.500	0.274	0.363	Al14
0.500	0.274	0.637	Al15
0.500	0.726	0.363	Al16
0.000	0.284	0.500	Al17
0.000	0.716	0.500	Al18
0.500	0.784	0.000	Al19
0.500	0.216	0.000	Al20
0.000	0.000	0.000	Al21
0.500	0.500	0.500	Al22

https://doi.org/10.3390/ma19040701
